# Potential value of saline-induced Pd/Pa ratio in patients with coronary artery stenosis

**DOI:** 10.3389/fcvm.2022.1001833

**Published:** 2023-01-06

**Authors:** Hiroyuki Kiriyama, Arihiro Kiyosue, Shun Minatsuki, Takuya Kawahara, Susumu Katsushika, Tatsuya Kamon, Kazutoshi Hirose, Hiroki Shinohara, Mizuki Miura, Akihito Saito, Hironobu Kikuchi, Satoshi Kodera, Masaru Hatano, Jiro Ando, Masahiro Myojo, Nobuhiko Itoh, Keisuke Yamamoto, Hiroshi Ikenouchi, Norifumi Takeda, Issei Komuro

**Affiliations:** ^1^Department of Cardiovascular Medicine, The University of Tokyo Hospital, Tokyo, Japan; ^2^Department of Cardiology, Moriyama Memorial Hospital, Tokyo, Japan; ^3^Biostatistics Division, Clinical Research Promotion Center, The University of Tokyo Hospital, Tokyo, Japan; ^4^Department of Cardiology, Kanto Central Hospital of the Mutual Aid Association of Public School Teachers, Tokyo, Japan; ^5^Department of Cardiology, Japanese Red Cross Medical Center, Tokyo, Japan

**Keywords:** saline-induced Pd/Pa ratio, resting full-cycle ratio, fractional flow reserve, epicardial coronary artery, physiological assessment

## Abstract

**Background:**

Fractional flow reserve (FFR) is the current gold standard for identifying myocardial ischemia in individuals with coronary artery stenosis. However, FFR is not penetrated as much worldwide due to time consumption, costs associated with adenosine, FFR-related discomfort, and complications. Resting physiological indexes may be widely accepted alternatives to FFR, while the discrepancies with FFR were found in up to 20% of lesions. The saline-induced Pd/Pa ratio (SPR) is a new simplified option for evaluating coronary stenosis. However, the clinical implication of SPR remains unclear.

**Objectives:**

In the present study, we aimed to compare the accuracies of SPR and resting full-cycle ratio (RFR) and to investigate the incremental value of SPR in clinical practice.

**Methods:**

In this multicenter prospective study, 112 coronary lesions (105 patients) were evaluated by SPR, RFR, and FFR.

**Results:**

The overall median age was 71 years, and 84.8% were men. SPR was correlated more strongly with FFR than with RFR (*r* = 0.874 vs. 0.713, respectively; *p* < 0.001). Using *FFR* < 0.80 as the reference standard variable, the area under the receiver-operating characteristic (ROC) curve for SPR was superior to that of RFR (0.932 vs. 0.840, respectively; *p* = 0.009).

**Conclusion:**

Saline-induced Pd/Pa ratio predicted FFR more accurately than RFR. SPR could be an alternative method for evaluating coronary artery stenosis and further investigation including elucidation of the mechanism of SPR is needed (225 words).

## Introduction

Evaluating the severity of coronary artery stenosis functionally, rather than angiographically, is currently of utmost importance. Measurement of the fractional flow reserve (FFR) by pharmacologically inducing maximal hyperemia is widely considered the best practice for invasive assessment of epicardial coronary stenosis. Revascularization under the FFR with a cut-off value of 0.8 has improved clinical outcomes (1–4) and is strongly recommended (Class I) in the latest guidelines in the U.S. and the Europe (5, 6); however, the FFR utilization rate has remained low worldwide (7), and possible reasons for the low adoption rate might include time consumption to measure FFR, costs associated with adenosine, complications, and FFR-related discomfort like flushing, chest pain, palpitations, hypotension, and so on (7).

Non-hyperaemic resting measurements that can prevent or compensate for the disadvantages of FFR are increasingly being developed; two large-scale randomized controlled trials have shown that revascularization strategies guided by instantaneous wave-free ratio (iFR) are non-inferior to those guided by FFR with respect to the rate of major adverse cardiac events at 1-year follow-up (8, 9). In addition to iFR, several resting physiological indexes that can be used as invasive tools to guide interventional strategy with similar clinical outcome have also been reported (10). The VALIDATE RFR study found that the resting full-cycle ratio (RFR), which measures the maximal relative pressure difference between diastolic pressure (Pd) and aortic pressure (Pa) (Pd/Pa) during the entire cardiac cycle, is diagnostically equivalent to iFR (11). However, despite the usefulness of resting indexes, discrepancies with FFR were found in up to 20% of patients in clinical settings (11, 12). At this time, resting indexes from 0.86 to 0.93 are considered in the “grey zone,” requiring further FFR measurement to determine whether there is myocardial ischemia or not (13), which complicates the process of evaluating myocardial ischemia.

Recently, intracoronary saline injection is getting some attention for the assessment of coronary physiology. Several pre-clinical and clinical studies have reported the induction of myocardial hyperemia by intracoronary saline injection using a dedicated catheter that injects saline through four side holes (14–19), although the mechanism is not yet fully understood. Furthermore, intracoronary bolus administration of saline can be applied to evaluate coronary microvascular function to know the mechanisms of not only coronary artery disease but non-obstructive coronary artery disease (20, 21).

Saline-induced Pd/Pa ratio (SPR) has been very recently reported as a means of predicting the functional significance of coronary stenosis assessed using FFR (22, 23), in which the Pd/Pa ratio is determined after injecting saline into the target coronary artery using diagnostic or guiding catheters, instead of adenosine injection. One of the advantages of SPR is its simplicity; it requires no drugs and can be performed in a short time. However, its usefulness remains unclear. In the present study, we aimed to compare the prediction accuracies of SPR and RFR based on FFR measurement, and to evaluate the usefulness of SPR compared with RFR in daily clinical practice.

## Materials and methods

### Study protocol

This was a multicenter prospective interventional study to evaluate the accuracy of SPR. We enrolled patients who were over 20 years old, had coronary artery disease, had undergone coronary angiography, and were judged as having 50% or more coronary stenosis by two experienced interventional cardiologists. Exclusion criteria were: (1) Severe valvular disease; (2) acute decompensated heart failure; (3) extreme bradycardia (heart rate <40 beats per minute); (4) allergy to adenosine; (5) life-threatening co-morbidities such as acute liver injury and renal disorder; (6) coronary total occlusion; and (7) judged ineligible for participation in this study by the responsible doctor. When conducting the analysis, lesions with 90% or more were excluded because of the concern about overestimating the result. Detailed inclusion and exclusion criteria are shown in Supplementary Table 1. The registration period was from February 2019 to June 2021.

### Ethics approval statement

This study involving human participants were reviewed and approved by a central-International Review Board in the University of Tokyo Hospital and the Ethics Committees in Kanto Central Hospital of the Mutual Aid Association of Public School Teachers and Japanese Red Cross Medical Center. It was conducted in accordance with the principles of the Declaration of Helsinki. It was registered in the UMIN Clinical Trial Registry (UMIN000039397). The patients and participants provided their written informed consent to participate in this study. The methods were carried out in accordance with approved guidelines.

### Coronary angiography and pressure measurements

**FIGURE 1:**
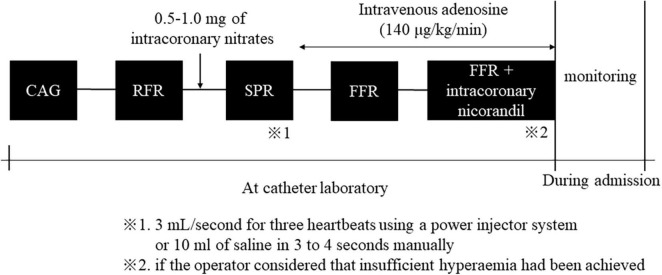
Outline of pressure measurements protocol. CAG, coronary angiography; RFR, resting full-cycle ratio; SPR, saline-induced Pd/Pa ratio; FFR, fractional flow reserve.

Conventional coronary angiography was performed using standard techniques *via* a transradial, transbrachial, or transfemoral approach (4–6 Fr). Coronary artery stenosis, defined as 50% or more stenosis, was visually diagnosed by two independent experienced interventional cardiologists at each institution. A physiological study using a pressure wire (Pressure Wire Aeris™ or Pressure Wire X™; Abbott^®^, USA) was also performed to assess the severity of coronary artery lesions. Hemodynamic measurements, including heart rate and aortic blood pressure, were recorded continuously throughout this procedure. Initially, the RFR was measured after the pressure wire had been advanced into the target coronary artery. After measurement of RFR, 0.5–1 mg nitrates were injected into the target coronary artery. More than 1 min after intracoronary nitrates, we evaluated SPR immediately after injecting an intracoronary bolus of saline at room temperature at 3 mL/s for three heartbeats through the catheter using a power injector system or 10 ml of saline in 3–4 s manually. SPR was defined at the inflection point between rapid increase and plateau. Finally, we evaluated FFR during peak hyperemia induced by intravenous infusion of adenosine (140 μg/kg/min). If the operator considered that insufficient hyperemia had been achieved, the FFR was measured again after intracoronary administering 2 mg of nicorandil. We monitored the patient’s symptoms and any complications during and after these procedures. Outline of pressure measurements protocol is shown in Figure 1.

### Other variables

Patient characteristics, past medical history, clinical features, prescribed medications, and laboratory data were collected from medical records.

### Statistical analysis

Categorical variables are presented as numbers and percentages, and continuous variables are presented as the median and interquartile range. Spearman’s rank correlation coefficient (*r*) was used to assess correlations between SPR and FFR and between RFR and FFR. We used Bland–Altman plots and 95% limits of agreement to analyze agreement between SPR and FFR. Receiver-operating characteristic (ROC) and area under the curve (AUC) analysis was performed to estimate the diagnostic performance of SPR and RFR. Since *FFR* < 0.80 is considered as the cut-off value to consider the revascularization, the cut-off value for the FFR thresholds is set at 0.80 (1–4). The sensitivity and specificity for the cut-off points of 0.86 and 0.93 for RFR were calculated, these being the gray zone limits for SPR (13). The Delong test was used to compare the areas under two correlated ROC curves (24). A *P*-value of < 0.05 was considered to indicate a statistically significant difference. We performed statistical analyses using JMP Pro version 16.0 statistical software (SAS Institute, Cary, NC, USA).

## Results

### Baseline characteristics

We enrolled 122 coronary artery lesions (114 patients) and excluded 10 lesions with severe stenoses with 90% or more. Finally, a total of 112 coronary artery lesions (105 patients) were evaluated during the study period. Baseline characteristics of the study cohort are shown in Table 1. The overall median age was 71 years and 84.8% were men. Target lesions in the left anterior descending artery, left circumflex artery, and right coronary artery were 74 (66.1%), 16 (14.3%), and 22 (19.6%), respectively. There were no fatal adverse effects in all procedures.

**TABLE 1:** Baseline characteristics.

Variable	Overall patients (*n* = 105)
***General***	
Age, year	71 (59–77)
Man, gender	89 (84.8%)
Height, cm	165.6 (158.3–170.0)
Weight, kg	65.8 (57.0–74.0)
BMI, kg/m^2^	24.4 (22.0–27.0)
Smoking	56 (53.3%)
Family history	17 (16.2%)
***Past Medical History***	
Hypertension	78 (74.3%)
Dyslipidemia	79 (75.2%)
Diabetes mellitus	52 (49.5%)
Chronic kidney disease	23 (21.9%)
Previous myocardial infarction–LAD/LCx/RCA	6 (5.7%)/1 (1.0%)/5 (4.8%)
Previous PCI–LAD/LCx/RCA	31 (29.5%)/15 (14.3%)/16 (15.2%)
***Clinical features***	
ACS	1 (1.0%)
eAP	50 (47.6%)
SMI	52 (49.5%)
Others	2 (1.9%)
***Medication***	
Calcium blocker	52 (49.5%)
Uric acid	11 (10.5%)
Nicorandil	10 (9.5%)
RAS inhibitor	60 (57.1%)
β blocker	41 (39.0%)
Statin	63 (60.0%)
Other antidyslipidemic drug	15 (14.3%)
Antiplatelet drug	77 (73.3%)
Anticoagulant drug	18 (17.1%)
Glucose lowering therapy	30 (28.6%)
Insulin	11 (10.5%)
**Procedure (lesions *n* = 112)**	
Target vessel: LAD/LCx/RCA	74 (66.1%)/16 (14.3%)/22 (19.6%)
Guide catheter: 4Fr/5Fr/6Fr	6 (5.4%)/99 (88.4%)/7 (6.3%)
Injection type: Manual/automatic	108 (96.4%)/4 (3.6%)
***Laboratory data***	
LDL-cholesterol, mg/dL	86.0 (68.0–109.3)
HDL-cholesterol, mg/dL	51.5 (41.8–62.1)
Triglyceride, mg/dL	138.0 (89.0–224.0)
HbA1c, %	6.2 (5.7–7.0)
Hemoglobin, g/dL	13.5 (12.8–15.0)
Hematocrit, %	41.4 (37.7–44.8)
Creatinine, mg/dL	0.86 (0.74–1.02)
eGFR, mL/min/1.73 m^2^	64.7 (54.4–75.5)
BNP, pg/mL	40.1 (13.5–68.3)

Data are expressed as number (percentage) or the median and interquartile range (IQR). BMI, body mass index; LAD, left anterior descending artery; LCx, left circumflex artery; RCA, right coronary artery; PCI, percutaneous coronary intervention; ACS, acute coronary syndrome; eAP, effort angina pectoris; SMI, silent myocardial ischemia; RAS, renin-angiotensin system; LDL-cholesterol, low density lipoprotein cholesterol; HDL-cholesterol, high density lipoprotein cholesterol; HbA1c, hemoglobin A1c; eGFR, estimated glomerular filtration rate; BNP, brain natriuretic peptide.

### SPR, RFR, and FFR

**FIGURE 2:**
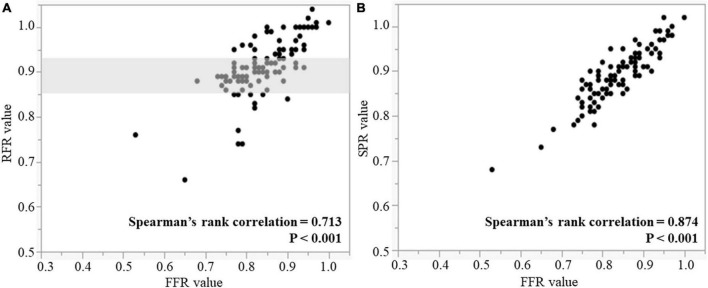
Correlations between RFR and FFR **(A)**, and SPR and FFR **(B)**. Gray shaded area is the RFR gray zone of 0.86–0.93. RFR, resting full-cycle ratio; SPR, saline-induced Pd/Pa ratio; FFR, fractional flow reserve.

**FIGURE 3:**
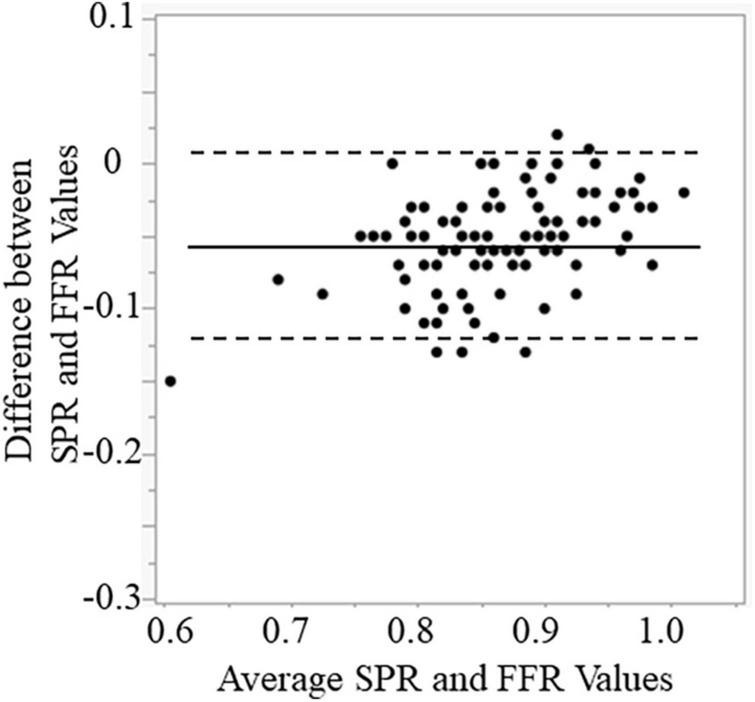
Bland–Altman plots showing agreement between SPR and FFR. SPR, saline-induced Pd/Pa ratio; FFR, fractional flow reserve.

**FIGURE 4:**
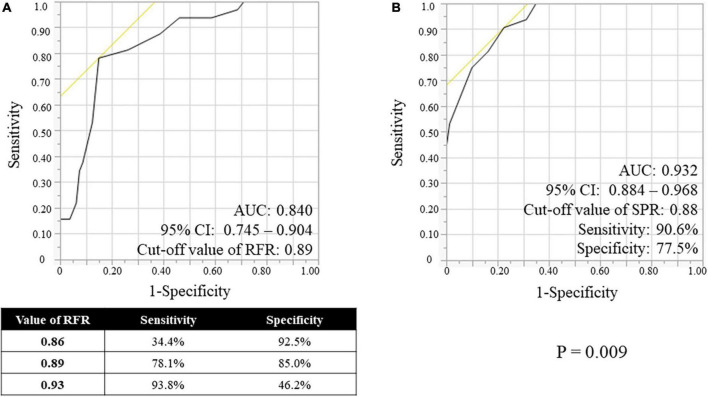
Receiver-operating characteristic (ROC) curves of the RFR **(A)** and the SPR **(B)**, using *FFR* < 0.80 as the reference standard variable. RFR, resting full-cycle ratio; SPR, saline-induced Pd/Pa ratio; FFR, fractional flow reserve; AUC, area under the curve; CI, confidential interval.

RFR, SPR, and FFR values are shown in Table 2. The median values and interquartile range of RFR, SPR and FFR were 0.91 (0.89–0.95), 0.90 (0.86–0.94), and 0.85 (0.79–0.89). Both scatter plots in Figure 2 shows that RFR and SPR were strongly correlated with FFR, but SPR was more strongly correlated with FFR than with RFR (Spearman’s rank correlation = 0.874 vs. 0.713, respectively; *p* < 0.001). Sixty-one lesions (54.5%) were in the gray zone of RFR from 0.86 to 0.93 (gray area in Figure 2A). Figure 3 shows the results of Bland–Altman analysis, which revealed strong agreement SPR and FFR (*p* < 0.001). The ROC curve for SPR, using *FFR* < 0.80 as the reference standard variable, showed the AUC was superior to that for RFR (0.932 vs. 0.840, respectively; *p* = 0.009) (Figure 4). The optimal cut-off value for RFR was 0.89 for prediction of *FFR* < 0.80 with sensitivity and specificity of 78.1 and 85.0%, respectively. The sensitivity and specificity of the gray zone threshold for RFR were 34.4 and 92.5%, respectively, in 0.86 of RFR and 93.8 and 46.2%, respectively, in 0.93 of RFR. The optimal cut-off value for SPR was 0.88 for prediction of *FFR* < 0.80 with sensitivity and specificity of 90.6 and 77.5%, respectively.

**TABLE 2:** Values of RFR, SPR, and FFR.

Variable	Overall lesions (*n* = 112)
RFR	0.91 (0.89–0.95)
SPR	0.90 (0.86–0.94)
FFR	0.85 (0.79–0.89)

Data are expressed as the median and interquartile range (IQR). RFR, resting full-cycle ratio; SPR, saline-induced Pd/Pa ratio; FFR, fractional flow reserve.

### Incremental value of SPR in the gray zone of RFR

**FIGURE 5:**
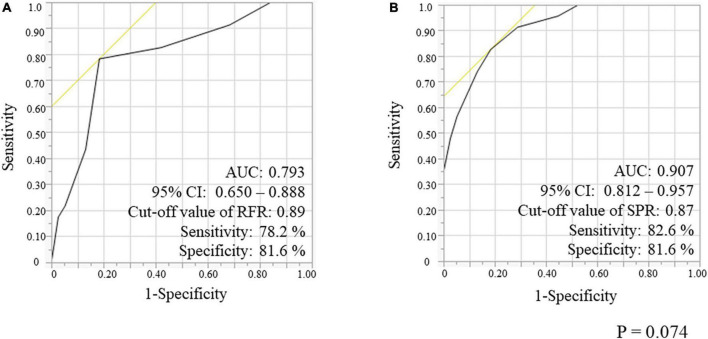
Receiver-operating characteristic (ROC) curves for RFR **(A)** and SPR **(B)**, using *FFR* < 0.80 as the reference standard variable in the gray-zone from 0.86 to 0.93 of RFR. RFR, resting full-cycle ratio; SPR, saline-induced Pd/Pa ratio; FFR, fractional flow reserve; AUC, area under the curve.

We evaluated the ROC curves for RFR and SPR in a lesion subgroup with gray zone of RFR consisting of 61 lesions (54.5%) (Figure 5). The AUC for RFR and SPR was 0.793 and 0.907, respectively, and the AUC value for SPR was higher than that for RFR (*p* = 0.074). The cut-off value for RFR was 0.89 with sensitivity and specificity of 78.2 and 81.6%, respectively, whereas the cut-off value for SPR was 0.87 with sensitivity and specificity of 82.6 and 81.6%, respectively.

## Discussion

In this study, we aimed to clarify the potential value of SPR for patients with coronary artery stenosis. There were two key findings. First, there was a significant correlation between SPR and FFR values, and the relationship was significantly stronger than that between RFR and FFR values. Second, the ability of SPR to correctly predict ischemia with *FFR* < 0.80 was significantly greater than RFR. In particular, similar results were obtained in the range from 0.86 to 0.93 of RFR, known as the gray zone.

Despite strong recommendations in US and European guidelines according to much evidence for FFR (5, 6), the use of FFR in daily practice is not sufficient enough. Penetration rate of FFR in many countries is reported to be less than 6% (7), which is unfortunately far from the strong recommendation of the guidelines. The reasons for this should be multifactorial including increased time and cost, discomfort and complications from FFR (7). Thus, resting physiological indexes are becoming more widely adopted due to their simplicity and large randomized evidence showing their usefulness (8, 9). However, discordance between FFR and resting indexes occurs in up to 20% of all cases in clinical settings (11, 12, 25); Svanerud et al. reported that the discordance of RFR and FFR was observed in 19% lesions (11), and in the present study, there was discordance between FFR and RFR in 23.0% lesions. Thus, these indicate that resting indexes may not serve as adequate substitutes for FFR.

Coronary physiology indexes other than resting indexes have been developed. In the CANICA study including 335 cases presenting intermediate coronary stenosis ranging (30–70%), the cut-off value of Pd/Pa ratio obtained immediately after intracoronary infusion of nitroglycerine (Pd/Pa-NTG) >0.88 had a high negative predictive value of 96.2% and sensitivity of 95% for *FFR* > 0.8 (26). In the CONTRAST study including 763 patients, the contrast medium Pd/Pa ratio (cFFR), in which suboptimal hyperemia is induced by infusion of contrast medium into the target coronary artery, is superior to resting Pd/Pa ratio and iFR for predicting FFR (85.8% accuracy vs. 78.5 and 79.9% for Pd/Pa ratio and iFR) (27). In the MEMENTO-FFR study, a cFFR/FFR hybrid approach showed a significantly lower number of lesions requiring adenosine than a resting Pd/Pa/FFR hybrid approach (28). Furthermore, intracoronary nicorandil (Nicorandil FFR) has also been proposed as an alternative hyperaemic agent to adenosine for FFR (29, 30). The value of each method excepting Nicorandil FFR was higher than that indicated by FFR. These results might imply the partial coronary hyperemia due to infusion of drugs, but further investigation is needed for the mechanisms. Those previous results suggested that each physiological assessment has a certain degree of accuracy for predicting functional ischemia and could assist to determine whether the lesion should be intervened. However, these coronary physiology indexes require infusion of drugs or other substances and have not been fully accepted as alternatives to the current standard techniques, such as FFR, and resting indexes.

Saline-induced Pd/Pa ratio is a type of physiological assessment method that does not require injection of any drugs other than saline into the target coronary artery; its usefulness has been investigated in several studies. Fujimori et al. investigated the accuracy of SPR for predicting *FFR* < 0.80 using 137 coronary lesions with over 50% angiographic diameter stenosis and found that the diagnostic performance of the cut-off 0.84 was specificity 94.3% and sensitivity 79.9% (22). Sato et al. also assessed the utility of SPR compared with FFR in 70 modest lesions (exceeding 30%) and reported excellent accuracy with a specificity of 98.2% and a sensitivity of 90.6% (23). The present study is the multicenter prospective study to perform RFR, SPR, and FFR successively at one time in patients with coronary artery stenosis (exceeding 50%), and our data also indicate that SPR is superior to RFR in predicting *FFR* < 0.80, consistent with previous reports (22, 23). Further, SPR has another advantage in terms of its convenience which requires a short time only about 10 s, therefore SPR could be an alternative method for evaluating coronary artery stenosis.

Currently, several basic and clinical research reported the intracoronary saline injection could induce myocardial hyperemia. De Bruyne et al. proposed that continuous intracoronary saline infusion at room temperature at a flow rate ≥15 mL/min using a dedicated catheter that injects saline through side holes could induce vasodilation of downstream resistance arteries and coronary hyperemia due to endothelial production of NO by hitting the vascular wall, decreasing local arterial oxygen content, myocardial ischemia (14). Adjedj et al. also reported infusion of saline at 20 mL/min using the same catheter could induce similar myocardial hyperemia using twenty open chest pigs and concluded epicardial wall vibrations might elicit myocardial hyperemia because the vasodilation was not related to the composition, the temperature of the indicator and the endothelial mediation in the study (16), and Gallinoro et al. claimed that local hemolysis was a potential mechanism of saline-induced hyperemia by saline infusion at 10 mL/min as rest and at 20 mL/min as hyperemia (17). Although the mechanism of saline-induced hyperemia is still not fully elucidated, it is becoming accepted that the methodology of saline-induced hyperemia could be measured at rest and during hyperemia at infusion rates of 10 ml/min and 20 ml/min for the LAD, and at slightly lower volume (infusion rates of 8 ml/min and 15 ml/min) for other coronary arteries with a dedicated catheter (14–19). In the present study, we evaluated SPR using diagnostic or guiding catheters, not a dedicated catheter, injecting saline at 3 mL/s for three heartbeats using a power injector system or 10 ml of saline in 3–4 s manually. The value of SPR was higher than those obtained with previous reports (22, 23), but the injection speed was much faster than in previous reports with a maximum of 20 ml/min (14–19), and the mechanism of saline administration through the side holes hypothesized in the previous studies might have partially functioned. Moreover, Fujimori et al. suggested that a low viscosity effect induced by intracoronary saline infusion might be considered as one possible mechanism of SPR (22), but further investigation to elucidate the mechanism of SPR is needed.

### Limitations

The present study has several limitations. First, the study cohort was relatively small despite this being a multicentre prospective study. Second, the cut-off value for SPR to predict *FFR* < 0.80 in the present study was 0.88, which differs from that reported by previous Japanese studies (22, 23). The results were derived from studies using small sample sizes, and thus the validity and usefulness of SPR needs to be further examined in other settings and also in large-scale studies. Third, different sizes of guiding catheter (4–6 Fr) were used, infusing the same amount of saline, which might have affected the values of SPR. However, Fujimori et al. suggested that no matter which guiding catheter (4–6 Fr) is used, there is a good correlation with the values of SPR. Fourth, the saline injection protocol is not standardized. We used diagnostic or guiding catheters as the study of Fujimori et al. (22), but allowed the examiners manual injection or power injection system as the manners of saline injection, which could have caused errors among the examiners. Furthermore, steady-state hyperemia is important in functional ischemia assessment and dedicated catheters were used for saline hyperemia in previous studies (14–19). In this study, we used diagnostic or guiding catheters and steady-state hyperemia might not be obtained with our protocol. Fifth, there is no quantitative coronary angiography (QCA) evaluation of coronary stenosis, lacking a detailed assessment of stenosis in the target coronary artery, however, the severity of stenosis was subjectively evaluated by two experienced interventionalists who performed the coronary angiography.

## Conclusion

Saline-induced Pd/Pa ratio predicted FFR more accurately than RFR. SPR could be an alternative method for evaluating coronary artery stenosis and further investigation including elucidation of the mechanism of SPR is needed.

## Data availability statement

The raw data supporting the conclusions of this article will be made available by the authors, without undue reservation.

## Ethics statement

The studies involving human participants were reviewed and approved by a central International Review Board in the University of Tokyo Hospital and the Ethics Committees in Kanto Central Hospital of the Mutual Aid Association of Public School Teachers and Japanese Red Cross Medical Center. It was conducted in accordance with the principles of the Declaration of Helsinki. It was registered in the UMIN Clinical Trial Registry (UMIN000039397). The patients and participants provided their written informed consent to participate in this study. The methods were carried out in accordance with approved guidelines. The patients/participants provided their written informed consent to participate in this study.

## Author contributions

HKir: conceptualization, methodology, validation, and writing—original draft, review, and editing. AK and SM: conceptualization, methodology, validation—review, and editing. TKaw: statistical revision. SKa, TKam, KH, HS, MMi, AS, HKik, MMy, and KY: data collection. SKo, MH, JA, NI, HI, NT, and IK: supervision. All authors contributed to the article and approved the submitted version.
