# Supplementary material for: Potential value of saline-induced Pd/Pa ratio in patients with coronary artery stenosis
Source: Front Cardiovasc Med. 2023 Jan 6;9:1001833. doi: 10.3389/fcvm.2022.1001833 (PMC9853169; doi:10.3389/fcvm.2022.1001833)
Supplement: Supplementary file 1 [file Table_1.DOCX]

| **Inclusion criteria** | **Primary registration** Patients with suspicion of coronary artery stenosis who receive coronary angiography  **Secondary registration** 1) Patients with coronary artery disease who undergo coronary angiography and judged having stenosis with 50% or more by 2 interventionists (at least one of them including specialists in Japanese Association of Cardiovascular Intervention and Therapeutics) 2) No particular limitation of the lesion site (left anterior descending artery, left circumflex branch, right coronary artery), history of PCI, and lesion form (in stent restenosis, calcified lesion) 3) Over 20 years old 4) Patients obtained document consents by the patient's will with sufficient understanding after receiving sufficient explanation for the participation of this study |
| --- | --- |
| **Exclusion criteria** | 1) Patients with severe valvular disease 2) Patients with decompensated heart failure 3) Patients with extreme bradycardia (HR <40 beats per minute) 4) Patients who cannot use adenosine necessary for FFR 5) Patients with co-morbidities such as acute liver injury and renal disorder that have serious effects on life 6) coronary total occlusion 7) Patients who are judged inappropriate for participation in this study by the responsible doctor |

**Supplemental table 1. inclusion and exclusion criteria**

PCI, percutaneous coronary intervention; HR, heart rate; FFR, fractional flow reserve.
